# The Role of Ghrelin, Salivary Secretions, and Dental Care in Eating Disorders

**DOI:** 10.3390/nu4080967

**Published:** 2012-08-13

**Authors:** Takakazu Yagi, Hirotaka Ueda, Haruka Amitani, Akihiro Asakawa, Shouichi Miyawaki, Akio Inui

**Affiliations:** 1 Department of Orthodontics, Medical and Dental Hospital, Kagoshima University, 8-35-1 Sakuragaoka, Kagoshima 890-8520, Japan; Email: tyagi@dent.kagoshima-u.ac.jp (T.Y.); ueda@dent.kagoshima-u.ac.jp (H.U.); 2 Department of Psychosomatic Internal Medicine, Kagoshima University Graduate School of Medical and Dental Sciences, 8-35-1 Sakuragaoka, Kagoshima 890-8520, Japan; Email: amitani@m3.kufm.kagoshima-u.ac.jp (H.A.); asakawa@m2.kufm.kagoshima-u.ac.jp (A.A.); 3 Department of Orthodontics and Dentofacial Orthopedics, Kagoshima University Graduate School of Medical and Dental Sciences, 8-35-1 Sakuragaoka, Kagoshima 890-8544, Japan; Email: miyawaki@dent.kagoshima-u.ac.jp

**Keywords:** anorexia nervosa, bulimia nervosa, ghrelin, salivary secretions

## Abstract

Eating disorders, including anorexia and bulimia nervosa, are potentially life-threatening syndromes characterized by severe disturbances in eating behavior. An effective treatment strategy for these conditions remains to be established, as patients with eating disorders tend to suffer from multiple relapses. Because ghrelin was originally discovered in the stomach mucosa, it has been widely studied over the past decade in an effort to uncover its potential roles; these studies have shed light on the mechanism by which ghrelin regulates food intake. Thus, studying ghrelin in the context of eating disorders could improve our understanding of the pathogenesis of eating disorders, possibly resulting in a promising new pharmacological treatment strategy for these patients. In addition, early detection and treatment of eating disorders are critical for ensuring recovery of young patients. Oral symptoms, including mucosal, dental, and saliva abnormalities, are typically observed in the early stages of eating disorders. Although oral care is not directly related to the treatment of eating disorders, knowledge of the oral manifestations of eating disorder patients may aid in early detection, resulting in earlier treatment; thus, oral care might contribute to overall patient management and prognosis. Moreover, ghrelin has also been found in saliva, which may be responsible for oral hygiene and digestion-related functions. This review discusses the pharmacological potential of ghrelin in regulating food-intake and the role of saliva and oral care in young patients with eating disorders.

## 1. Introduction

Eating disorders, including anorexia nervosa (AN) and bulimia nervosa (BN), are serious diseases that primarily affect individuals especially in late adolescence and young adulthood. These disorders are typically life-threatening if proper treatment is not administered. Patients with eating disorders are diagnosed based on criteria of the *Diagnostic and Statistical Manual of Mental Disorders 4th Edition Text Revision* (DSM-IV-TR) published by the American Psychiatric Association [1]. AN is classified in the restricting subtype (AN-R) and the binge/purge subtype (AN-BP), whereas BN is classified in the binge/purge subtype (BN-BP) and the non-purging subtype (BN-NP). Moreover, approximately half of eating disorder cases are categorized as an eating disorder not otherwise specified (EDNOS). Although it is thought that AN and BN represent separate diseases, some bulimia patients are found to have a history of anorexia. These patients have an obsessive fear of gaining weight and an unrealistic perception of their current body weight. Longitudinal follow-up studies of anorexia and bulimia nervosa have found that a significant proportion of subjects change diagnostic status to another eating disorder and such diagnostic transitions need to be considered for classification of the disease [2,3,4]. 

Importantly, numerous individual therapeutic strategies for these diseases, including behavioral therapy, family therapy, psychotherapy, and pharmacotherapy, have been challenged; therefore, a multidisciplinary care plan that is appropriate to each pathological condition has been adopted [1]. However, a definitive treatment protocol remains to be established.

Because the ghrelin family was originally discovered in the endocrine X/A-like cells of the gastric mucosa in rats and humans [5], numerous researchers have studied these brain–gut-related neuropeptides. Remarkable progress has recently been made with respect to energy regulation [6,7,8]. Thus, ghrelin-based pharmacotherapy may be a promising approach for the treatment of patients with eating disorders. Ghrelin is the only hormone with an orexigenic effect following peripheral administration [7]. Ghrelin is responsible for various functions, including the stimulation of growth hormone secretion, gastric motility, and gastric acid secretion, as well as the induction of a positive energy balance [8].

Additionally, several studies have focused on the oral function of ghrelin, as the oral region plays an important role in food intake. The oral system has many digestive functions, including mastication and taste of food, salivary secretion for initial digestion, creating a food bolus, and swallowing of food. Therefore, oral dysfunction leads to decreased quality of life. In particular, human salivary glands, which can produce and release ghrelin, have several remarkable roles in oral function [9,10]. This review focuses on the function of ghrelin and the role of salivary secretions in patients with eating disorders.

## 2. Anorexia and Bulimia Nervosa

Epidemiological data from eating disorder patients have been significantly affected by methodological differences and the use of inconsistent criteria during data collection. Although the DSM classification is widely used worldwide, the DSM-III had been used to survey these populations until the DSM-IV-TR was published in 2000 [11]. Therefore, it is important to note that eating disorder epidemiological data might reflect the specific criteria used in a particular survey.

The published prevalence rate of AN ranges between 0% and 0.9% in young girls after a 2-stage screening approach, although most studies have reported substantially higher prevalence rates for EDNOS, including partial AN syndromes [12,13]. A partial syndrome is usually noted in purging disorder and binge eating disorder (BED) patients. In Europe, a survey based on DSM-IV criteria revealed an average prevalence rate of 0.29% for AN [12], with an incidence rate of 8.0 per 100,000 individuals per year. A study in Singapore reported that 65% of AN patients belonged to the AN-R subtype, while 35% belonged to the AN-BP subtype [14]. In clinically diagnosed Japanese female AN patients, the reported prevalence rates ranged from approximately 0.025% to 0.2% [15], and the ratio of AN-R patients was larger than that of AN-BP patients [16]. Of note, an upward trend in AN incidence has been observed since the 1970s [3]. The age of onset of the disorder is usually less than 25 years [3,12,17], and majority of the patients (95%) are female. The likelihood of recovery is less than 50% after 10 years, with approximately 25% of patients remaining ill, and the mortality rate varies between 0% and 25% [18]. Although the reason for the marked sex differences observed in the incidence of eating disorders remains unclear, an experiment in which a short fasting period was introduced to Swedish high school students demonstrated that compared to young men, young women found it more difficult to compensate for the lack of meal size [19]. Previous review articles have suggested that anxiety disorder, which is one of the most prevalent comorbid psychiatric disorders observed in AN patients, commonly predates the onset of the eating disorder and begins in childhood; furthermore, the increased occurrence of several psychic disturbances in teenagers and young adults is associated with responsiveness to hormonal changes (e.g., estrogen) [20,21]. Therefore, it may be that the drastic changes that occur in pubertal hormone secretion affect the pathophysiological onset of AN. Common symptoms in patients with AN include restrictive behavior, binge eating, purging behavior, excessive exercise, repeated body checking, body image disturbances, neuroticism, negative emotions, low self-esteem, and low cooperativeness. Many patients are also diagnosed with anxiety, depressive disorder, and obsessive–compulsive disorder [22]. These comorbidities in AN patients contribute to persistent food restriction and body image disturbances. However, the etiology of these multifactorial disorders is poorly understood [23]. While weight restoration of malnourished AN patients is manageable, relapse is a common problem [24]. Taken together, these findings indicate that AN is a chronic, severe disorder, and unfortunately, this distressing scenario has not changed over the past 50 years [18]. 

Some AN patients exhibit bulimic behavior, and most patients with BN have a history of AN [12]. Therefore, it would not be surprising if the prevalence of BN was similar to that of AN. Among patients with BN, 19% show insufficient intake, whereas 44% exhibit overconsumption [25]. After voracity, BN patients show signs of mental depression, vomiting, and laxative abuse due to feelings of guilt. Although the health situation for BN patients is considered less severe than that of AN, the average incidence of BN is approximately 12 per 100,000 people (13.5 in Rochester, MN, USA [26]; 11.5 in the Netherlands [12]; and 12.2 in the United Kingdom [27]), which is higher than the incidence rates of AN. The outcome for patients with BN is also poor [27,28]. BN onset occurs between the ages of 12–40 years, with the highest prevalence occurring at the ages of 15–30 years [29]; the average age of BN patients is higher than that of AN patients [12]. In Japan, it was reported that the prevalence rates for BN ranged from 1.9% to 2.9% [15], and the number of BN-BP patients was greater than that of BN-NP patients [16].

Patients with eating disorders may experience serious medical consequences, including detrimental effects on bone and oral health [30,31,32]. Eating disorder patients with binge/purge subtype who exhibit chronic and frequent self-induced vomiting show several oral symptoms, including mucosal atrophy, dental erosion, and swelling of the salivary glands, with accompanying pain [33]. Previous studies suggested that adolescent and young female AN patients commonly showed low bone mass data at the early stages of the disorder [34,35]. Moreover, these patients lose enthusiasm for eating under the aggravation of oral symptoms and tend to stop eating. As a result, they become malnourished, with increased risks of osteoporosis and bone fracture throughout their lives [36].

The basic treatment for AN and BN primarily consists of several therapies, such as behavioral and family therapy, personal psychotherapy, and pharmacological therapy. However, a definitive treatment protocol for these diseases is lacking. Recently, progress has been made in the development of pharmacological therapy involving neuropeptides [37]. Importantly, a proactive stance must be taken regarding secondary supportive oral care, as patients with eating disorders experience gradual deterioration of oral functions, defenses against oral diseases, and bone mass, due to malnutrition.

Therefore, a basic medical treatment strategy and secondary supportive oral care are necessary for the treatment of these eating disorders. Early detection and vigorous intervention with restoration to a normal medical state are effective in reducing the mortality associated with these disorders [38]. Fortunately, dental practitioners typically examine patients on a regular basis, and often for the duration of a child or adolescent’s lifetime, suggesting that dentists and hygienists play an important role in the early identification of these patients through their administration of oral care.

## 3. The Role of Ghrelin Gene Products in Anorexia and Bulimia Nervosa

### 3.1. Regulation of Food Intake by the Hypothalamic Neuropeptide Signaling Pathway

The etiology and pathophysiology of eating disorders suggest that a derangement of the feeding regulation network causes an aberration of eating behavior. The center of this network exists in the hypothalamus; therefore, an understanding of the interrelationship of the factors that regulate this feeding system might aid in improving eating disorder treatment. Previous studies have elucidated that the hypothalamic feeding regulation network consists of the ventromedial hypothalamus (VMH), the lateral hypothalamus (LH), the arcuate nucleus (ARC), and the paraventricular nucleus (PVN) [39]. Neuropeptide Y (NPY) and agouti-related protein neuron (AgRP) function to increase food intake, whereas proopiomelanocortin (POMC) and cocaine- and amphetamine-regulated transcript (CART) act to inhibit food intake in the ARC. In addition, both these types of neurons are affected by peripheral information [39,40,41]. Melanin-concentrating hormone (MCH) and orexin are expressed in the neurons of the LH. MCH and orexin establish synaptic contact with the ARC and regulate food intake [42]. Corticotrophin-releasing factor (CRF) mediates the emotional stress-induced inhibition of food intake in neurons of the PVN. CRF also establishes synaptic contact with the ARC [43].

Additionally, many hormones secreted by peripheral tissues have been shown to play a role in the regulation of appetite [37]. Several prospective studies examined the hormonal balance involved in feeding and fasting, and investigated the mechanism of feeding regulation in rats [5,6,44]. Ghrelin, secreted from the stomach, activates NPY/AgRP neurons and inhibits POMC/CART neurons; thus, ghrelin increases meal size. Other feeding regulatory hormones, including insulin, leptin, cholecystokinin (CCK), and peptide YY (PYY), induces the suppression of food intake [37]. These peripheral hormones influence the activity of NPY/AgRP and POMC/CART neurons of the ARC through the vagus nerve and solitary tract nucleus (NTS) [39]. Furthermore, ghrelin may directly or indirectly affect these neurons, because acyl ghrelin (the active form of ghrelin) is capable of crossing the blood–brain barrier [44,45]. These findings suggest that the hypothalamus, including the ARC, may be a major site for transducing afferent input from the circulating leptin, insulin, and ghrelin into a neuronal response.

**Figure 1:**
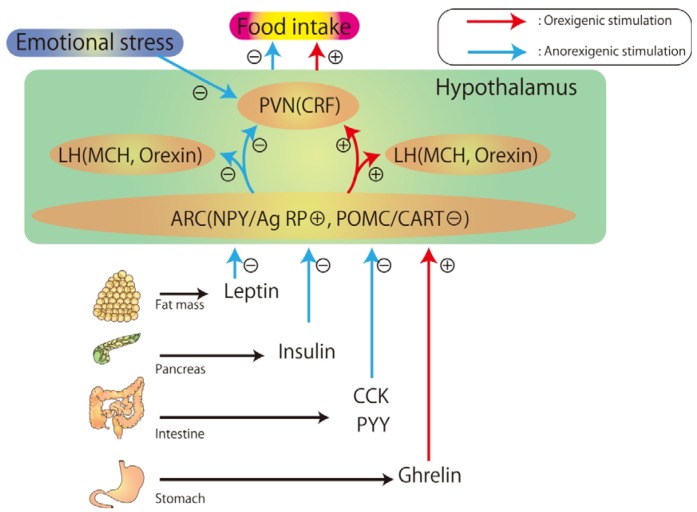
A simplified model of the feeding regulation hypothesis involving the hypothalamic neuropeptide signaling pathway. Leptin, insulin, CCK, and PYY stimulate an anorexigenic pathway and inhibit an orexigenic pathway. The effect of ghrelin in the hypothalamus is opposite to that of leptin, insulin, CCK, and PYY. Ghrelin stimulates an orexigenic pathway and inhibits an anorexigenic pathway. Together, this system functions to regulate food intake to optimize metabolic energy balance. NPY: neuropeptide Y; POMC: proopiomelanocortin; AgRP: agouti-related protein; CART: cocaine- and amphetamine-regulated transcript; ARC: arcuate nucleus; PVN: paraventricular nucleus; MCH: melanin-concentrating hormone; LH: lateral hypothalamus; CRF: corticotrophin-releasing factor.

Therefore, it is important to understand the effect of these hormones and neuropeptides participating in the hypothalamus in order to develop adequate pharmacotherapeutic approaches for patients with eating disorders. Ghrelin is a potentially useful hormone for medical treatment of food intake-related diseases. Figure 1 shows the feeding regulation hypothesis of the hypothalamic neuronal signaling pathway.

### 3.2. Ghrelin Gene Products

Ghrelin is an endogenous ligand of the growth hormone secretagogue receptor (GHS-R), which was discovered in the stomach. It is the first-identified orexigenic peptide of the peripheral tissues, and consists of a 28-amino acid peptide cleaved from the 117-amino acid precursor, preproghrelin [5,6]. Ghrelin is present in mammals and numerous other vertebrates [5,46,47,48] and has been reported to stimulate appetite and food intake in various diseases, including chronic heart failure, chronic obstructive pulmonary disease, and cancer [37]. In humans, ghrelin circulates in the peripheral blood and exists in 2 major molecular forms: acyl ghrelin, which has an n-octanoylated serine at position 3, and des-acyl ghrelin, which lacks n-octanoylation [49]; this fatty acid is thought to be the center of activity [50]. Both molecular forms are synthesized in the ARC [51,52,53,54] as well as in the stomach [55,56,57]. Acyl ghrelin is quite unstable and rapidly degrades to des-acyl ghrelin or even smaller fragments [58]. Acyl ghrelin acts on the GHS-R of the vagal afferent nerve in the stomach. Information from the solitary tract nucleus (NTS) is then projected to the ARC of the hypothalamus. Acyl ghrelin acts on GHS-R to stimulate GH release [59]. While acyl ghrelin induces a signal for mealtime hunger and meal initiation [37], it also affects body weight and adiposity [60]. In addition, acyl ghrelin induces a protective effect in the gastric mucosa [61] and is involved in the regulation of gastrointestinal motility [62,63]. Des-acyl ghrelin affects food intake [37,56], gut motility [64], body size development [56], adipogenesis [65], insulin secretion, and resistance to increased papillary muscle tension [66], as well as cell proliferation and survival [67]. Des-acyl ghrelin may block the orexigenic activity of acyl ghrelin [68]. Obestatin is the most recently discovered member of the ghrelin family; it mediates actions that are in opposition to those of ghrelin with regard to feeding and gastrointestinal motility [37]. Interestingly, a recent study showed that obestatin treatment suppressed body weight increases and gastric emptying in rats [69]. 

### 3.3. Ghrelin Abnormalities in Anorexia and Bulimia Nervosa

Although BN patients engage in vomiting and abnormal eating behaviors, some overlap may exists in the underlying etiologies of AN and BN [23]. Because chronic and recurrent abdominal discomfort and distension as well as fear of obesity commonly occur in AN patients, they are often unable to increase their food intake. As a result, abdominal discomfort is associated with chronic malnutrition, which induces functional and organic changes in the gastrointestinal tract [70]. Because ghrelin is involved in increasing food intake, examining the ghrelin level of patients with eating disorders may help to understand their eating abnormalities. 

Previous studies have shown that the total plasma ghrelin levels in AN patients (of both subtypes) are higher than those of controls [71,72,73,74,75,76,77,78,79,80,81,82,83,84,85,86,87]. In one study, acyl ghrelin levels were decreased after an oral glucose tolerance test in both subsets of AN patients and control subjects, whereas postprandial decline of plasma acyl ghrelin response to the oral glucose tolerance test was blunted in female patients with AN relative to control subjects [88]. Several studies showed that plasma des-acyl ghrelin levels are significantly higher in AN patients than in control, and plasma acyl ghrelin levels tend to be higher in AN patients than in control without a significant difference [88,89,90,91]. Moreover, the ratio of des-acyl ghrelin to acyl ghrelin in AN-R patients was higher than that of controls [88,89,90,91]. These results suggest that increased des-acyl ghrelin levels may prevent AN patients from eating and an insufficiently elevated acyl ghrelin level may contribute to the AN symptoms.

Plasma obestatin was also found to be significantly higher in AN-R patients, whereas they were decreased during increased food intake in patients with AN-R [75,88,92]. In addition, plasma obestatin levels were higher in AN patients than in thin, body mass index-matched women with normal obestatin levels [87]. The obestatin level decreased after an oral glucose tolerance test in both AN patient subgroups and control subjects [88]. Taken together, the results of these studies suggest that obestatin and acyl ghrelin might be nutritional markers, reflecting body adiposity and insulin resistance.

A previous study revealed that nutritional rehabilitation completely restored elevated plasma ghrelin to normal levels [79]. In addition, re-nutrition from successful treatment in AN patients restored normal insulin secretion and glucose responses to food ingestion [83]. Of note, intravenous administration of ghrelin in patients with AN-R improved epigastric discomfort and increased hunger sensations and food intake [93]. These results suggest that the plasma levels of ghrelin and obestatin are influenced by acute and long-term changes in energy homeostasis during severe emaciation or re-nutrition in patients with AN.

Interestingly, plasma ghrelin levels are also higher in patients with BN that in control subjects [74,94]. After modified sham feeding, circulating levels of ghrelin were significantly enhanced in patients with BN-P as compared to those in healthy controls, whereas circulating ghrelin levels were elevated in both groups immediately after eating [95]. Separate studies showed that both BN-P patients and healthy controls exhibited elevated ghrelin levels before meals, with reduced ghrelin suppression after eating [96,97]. However, normal endocrine and metabolic responses to acute ghrelin administration were observed in both BN-P patients and healthy women [98]. In light of these results, patients with BN may be characterized by exhibiting increased ghrelin secretion during the cephalic phase [95,98]. In addition, the mean plasma ghrelin level in BN-P was higher than that in both BN-NP and controls despite similar nutritional parameters such as body mass index, percent body fat and serum cholinesterase concentration [73]. There were significant correlations among plasma ghrelin values, frequencies of binge/purge cycles and serum amylase values for both AN-BP and BN-P patients [73]. These results suggest that habitual binge/purge behavior might have an influence on circulating plasma ghrelin levels in both BN-P and AN-BP.

In contrast, plasma obestatin levels were not increased in patients with BN [76], and the ratio of obestatin to total ghrelin was not significantly different between patients with BN and the control patients [76]. 

Although the mechanisms by which total ghrelin and obestatin levels become elevated in AN and BN patients remains unknown; randomized and large-scale investigations are necessary to confirm the efficacy of ghrelin treatment for eating disorders.

## 4. The Role of Salivary Secretions in Eating Disorders

### 4.1. Eating Disorders and Oral Health

Poor oral hygiene and poor health are common in patients with AN and BN [99,100]. Most oral manifestations caused by chronic vomiting, nutritional deficiencies, and consequent metabolic impairment result from direct or indirect causes in AN and BN patients [38]. In addition, oral manifestations are caused by lack of personal oral hygiene care, underlying psychological disturbances, modified nutritional habits, or drug use [38,101,102]. Typical oral symptoms associated with eating disorders include mucosa atrophy, tooth erosion, gingivitis, hyperesthesia, periodontitis, necrotic salivary gland dysplasia, salivary adenopathy, hypoptyalism, and xerostomia [38,101]. Because AN-BP patients exhibit bulimic behavior and BN manifestation and often go through a phase of AN, many oral symptoms are found in both AN and BN patients, particularly in patients of the binge eating/purging subtype with vomiting. Slight differences exist between the restricting subtype and binge eating/purging subtype with regard to oral symptoms. Oral symptoms that are directly related to chronic vomiting directly include dental erosion of the palatal surface [38,103] and mucosal atrophy [38]. Previous reports indicated that medications such as dextrose tablets and sucrose-containing vitamin C beverages increase the incidence of dental caries [104,105]. Although the association between the rate of development of caries and vomiting frequency remains unclear [106,107,108], this damage exposes the dentin and results in tooth hypersensitivity [109,110] and occlusal changes, such as an anterior open bite [104]. Oral symptoms that are indirectly related to chronic vomiting include generalized gingival swelling, spontaneous gingival bleeding, mucosal atrophy, marginal periodontitis, and gingivitis, which are affected by vitamin C deficiency and malnutrition [111,112]. Sialadenosis (non-inflammatory enlargement of the salivary glands) is a frequently occurring manifestation of eating disorders, which may be caused by peripheral autonomic neuropathy related to disordered metabolism and salivary secretions [38,113]. A reduction in the flow of saliva may be related to the side effects of drugs prescribed for the treatment of depression [114]. Of note, the frequent use of acidic sports drinks during physical activity or carbonated drinks to decrease the reflex hunger stimulus may cause labial dental erosion in patients with AN-R [115,116]. Necrotizing sialometaplasia has also been reported to be associated with BN [117]. 

Oral manifestations (e.g., sialadenosis, palatal erythema, and unexplained clinical oral symptoms) may be caused at an initial stage of disease onset [38]. Therefore, the subtle changes that occur in the oral regions may be utilized as early indicators of a serious underlying psychiatric condition. In Table 1, the main effects of oral manifestations are summarized.

**Table 1:** Oral manifestation of patients with anorexia nervosa (AN) and bulimia nervosa (BN).

Oral manifestations		AN-R	BN-BP
Mucosal lesions	Mucosal atrophy	+	+
	Erythematous lesions (soft palate)	−	+
Dental lesions	Dental erosions (labial surfaces)	+	
	Dental erosions (lingual and occlusal surfaces)		+
	Caries	+	+
	Dental sensitivity (hyperesthesia)		+
Periodontal lesions	Gingivitis	+	+
	Periodontitis	+	+
Salivary manifestations	Salivary glands tumefaction	+	+
	Hyposalivation	±	±
	Necrotizing sialometaplasia		+
Other oral symptoms	Xerostomia	+	+
	Glossodynia	+	+
	Oral burning sensation	+	+
	Dysgeusia	±	±
	Episodes of oral pain	+	+

### 4.2. The Importance of Salivary Secretions in Eating Disorder Patients

To protect eating disorder patients from the aforementioned clinical oral symptoms, saliva plays several important roles in the oral cavity. Saliva is secreted from 3 major paired glands (parotid, submandibular, and sublingual) and from hundreds of minor salivary glands spread over the majority of the oral mucosa. Salivary secretion is not induced by bite and/or bruxism alone [118,119]; it is also induced by mastication [120]. Profuse salivary secretion is primarily induced during eating, which is a salivary gland reflex controlled by the autonomic nervous system. A lesser amount of saliva (resting saliva or unstimulated saliva) is secreted that always covers the surface of the oral and pharyngeal cavities. In addition, a previous report indicated that salivary secretions and swallowing improved acidic pH in the esophagus during sleep [121]. Saliva is comprised of lysozyme, peroxidase, secretory immunoglobulin A (IgA), anti-microbial histamine, and mucin. Epidermal and transforming growth factors found in saliva promote tissue growth, differentiation, and wound healing [122]. It should be noted that acyl ghrelin and des-acyl ghrelin has been detected in human saliva as well. Ghrelin-positive cells have been also observed in the salivary glands. [9,123] However, further research is required to understand why ghrelin is produced in the salivary glands. Oral imbalances caused by decreased salivary flow may affect the motivation of a patient with an eating disorder to increase food intake [124].

### 4.3. Regulation of the Salivary Secretion System

Salivary secretion is controlled by the sympathetic and parasympathetic autonomic nervous systems. The parasympathetic nerve is related to the secretion of water and electrolytes, whereas the sympathetic nerve is related to the secretion of proteins by exocytosis from acinar cells [125]. As described above, the greatest amount of saliva is induced after food intake. This salivation response is initiated by various sensory inputs, including visual, olfactory, oropharyngeal, and esophageal senses (gustatory, mechanical, and thermal) [126]. Afferent nerve impulses from the salivary reflex pass to the salivary nuclei through the medulla oblongata, including the NTS and the parabrachial nucleus (PBN), and these impulses affect the parenchyma of the salivary glands from these centers via the efferent parasympathetic secretomotor nerves [125]. The integration of impulses from the primary salivary centers to the glands depends on central regulation. Retrograde labeling of neurons revealed that the primary parasympathetic salivary centers form connections with the LH and PVN, the central nucleus of the amygdala (CeA), and the PVN and preoptic area (POA) [127,128]. Both excitatory (gamma aminobutyric acid-containing) and inhibitory (glycine-containing) nerves appear to synapse in the salivary centers [127,128]. Because ghrelin is produced by the salivary glands, central regulation by the neural network on salivary secretion may affect its production in saliva. However, the presence of central neural connections between the primary salivary centers and other nuclei remains unclear, and further studies are required in this regard.

Although salivary secretions (which include sIgA, histamine, defensin, cytokines, growth factors, hormones, and mucin [129]) play a critical role in innate immunity and host defense at mucosal surfaces such as adaptive cytoprotection through prostaglandins in the stomach, ghrelin produced in oral cavity may also exhibit a regulatory role in innate immune responses to inflammatory infections [130].

Ghrelin levels in the saliva of obese children, of adolescents, and of young healthy subjects are correlated with serum ghrelin levels and body mass index [10,131]. These results suggest the possibility that the measurement of ghrelin levels in saliva is a non-invasive and generally preferred alternative method to plasma sampling for understanding disease conditions in eating disorder patients.

The stress response involves the activation of the hypothalamic–pituitary–adrenal (HPA) axis and the sympathetic nervous system. The HPA axis is highly activated in response to physiological or psychological stressors, and CRF, which stimulates the anterior pituitary gland to release adrenocorticotropin-releasing hormone (ACTH), is released from the hypothalamus. ACTH stimulates the release of cortisol from the adrenal glands [132]. Given that the pathogenesis of AN consists of both physiological and psychological components, CRF from both the hypothalamus and the amygdala is responsible for anorexic behavior as a function of stress [133]. Hypercortisolemia associated with an elevated CRF level is commonly observed in patients with AN who have protein-calorie malnutrition [134]. Compared to healthy controls, pre-stress levels of salivary cortisol were significantly enhanced in AN-R patients. However, salivary cortisol levels were not significantly different between BN-BP patients and healthy controls [135]. Under psychosocial stress and during the acute phase of their disorder, female patients with AN-R showed a normal cortisol response, while the level of salivary cortisol in BN-BP patients hardly showed a change [135,136]. In addition, salivary levels of α-amylase have also been reported to increase under physically and psychologically stressful conditions and are associated with norepinephrine (NE) changes in response to stress [137]. Recent research revealed that in pre-stress conditions, salivary α-amylase levels in AN-R patients were not significantly different, whereas those in BN-BP patients were enhanced as compared to those in healthy women. Under conditions of psychosocial stress, salivary α-amylase levels were significantly lower in AN-R patients than in controls, while those in BN-BP patients were not significantly different [135].

**Figure 2:**
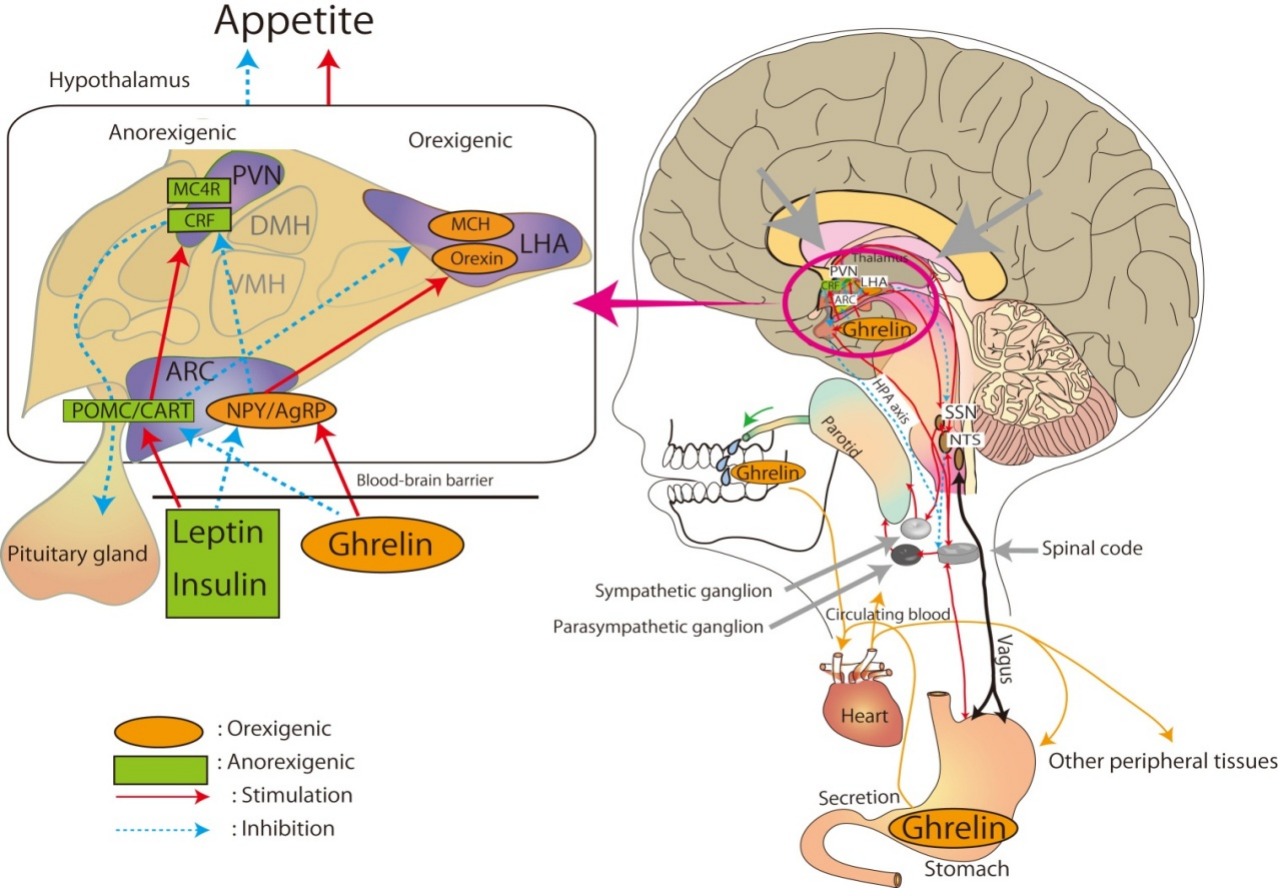
Feeding regulation of neuroendocrine hormones and salivary secretion. Salivary secretion is stimulated through the parasympathetic and sympathetic ganglia. The parasympathetic ganglion receives a stimulus from the nucleus of the solitary tract (NTS), and the sympathetic ganglion receives input from the NTS via the spinal cord. Both ganglia regulate salivary secretions. The parotid salivary glands supply ghrelin. Ghrelin circulates and spreads throughout the human body and brain. Ghrelin acts on specific target tissues. The function of ghrelin is mediated by the autonomic nervous system as well as the hypothalamic–pituitary endocrine axis. Ghrelin affects the metabolic regulation of food intake to achieve homeostasis. This hypothetical feeding regulation model suggests that ghrelin provides a promising pharmacotherapeutic approach for treating eating disorders. NPY: neuropeptide Y; POMC: proopiomelanocortin; AgRP: agouti-related protein; CART: cocaine- and amphetamine-regulated transcript; ARC: arcuate nucleus; PVN: paraventricular nucleus; MCH: melanin-concentrating hormone; LH: lateral hypothalamus; CRF: corticotrophin-releasing factor; MC4R: melanocortin 4 receptor; SSN: superior salivatory nucleus; NTS: nucleus tractus solitarii.

Therefore, salivary cortisol and α-amylase levels might also be used to estimate the degree of stress in patients with anorexia and bulimia. Figure 2 illustrates a portion of the salivary secretion system, the circulation of ghrelin, and the hypothetical hypothalamic feeding regulation. 

## 5. Treatment

Although AN is a chronic disorder that is resistant to treatment and prone to relapse, medical management of drug usage, behavioral therapy, family therapy, psychotherapy, outpatient therapy, and hospitalization are all necessary treatment modalities for young patients. However, this distressing scenario has remained unchanged in the past 50 years [18], and relapse is a serious problem [24]. Therefore, new treatment strategies are urgently needed. In the treatment of eating disorder patients, it is helpful to consider the following treatment objectives: (1) to eliminate the pattern of binge eating and compensatory behaviors in the early stage of the disorder; (2) to establish a more normal eating pattern with regular, balanced meals; (3) to address any physical complications of the illness, such as dental enamel erosion and abnormal salivary secretion; (4) to address the psychological issues that accompany the illness, including low self-esteem, body image dissatisfaction, and other dysfunctional thought patterns; (5) to address comorbid conditions such as mood disorders; and (6) to prevent relapse. To meet these objectives, disease management requires psychological, nutritional, and medical services provided by a multi-disciplinary team. A pharmacological approach is likely to be among the most effective treatments. Although selective serotonin reuptake inhibitor (SSRI) fluoxetine was suggested to be effective, this drug has shown limited efficacy in AN patients [138]. Moreover, combined with a multidisciplinary approach that includes nutritional rehabilitation and psychotherapy, adjunctive pharmacotherapy may be useful in addressing both the eating disorder psychopathology and comorbid psychiatric disorders [139]. However, recent evidence suggests that an effective pharmacotherapeutic strategy for AN has yet to be established. Recent studies have shown that the acyl ghrelin/des-acyl ghrelin obestatin balance could be essential for adaptation of the body to nutritional changes [75,88,140]. The aforementioned ghrelin gene products are likely to be part of the promising pharmacotherapeutic approach for achieving energy homeostasis in eating disorder patients.

Importantly, behavioral therapy is essential for effective, multidisciplinary treatment. The scientific basis for the suggestion that family-based treatment is effective in children with AN is based on the findings of a randomized, controlled trial [141] and a 5-year follow-up study [142]. However, another study showed that of 194 patients who underwent treatment, 54 (28%) dropped out or withdrew, 58 (30%) of 140 patients who completed the treatment went into remission after cognitive behavior therapy for BN, while 21 (44%) patients experienced relapse [143]. Therefore, treatment for eating disorders requires another effective therapy in addition to pharmacotherapeutic approaches.

With early detection and adequate treatment, the prognosis for recovery from eating disorders can be good [144]. Some studies show that oral problems associated with eating disorders can be manifested as early as 6 months after the individual consistently engages in eating behaviors involving serious disturbances [145,146]. Given that the onset of eating disorders is occurring increasingly earlier in childhood and can lead to a series of oral manifestations [32], the dentist may be the first healthcare provider to assess the physical and oral consequences of eating disorders in children.

**Figure 3:**
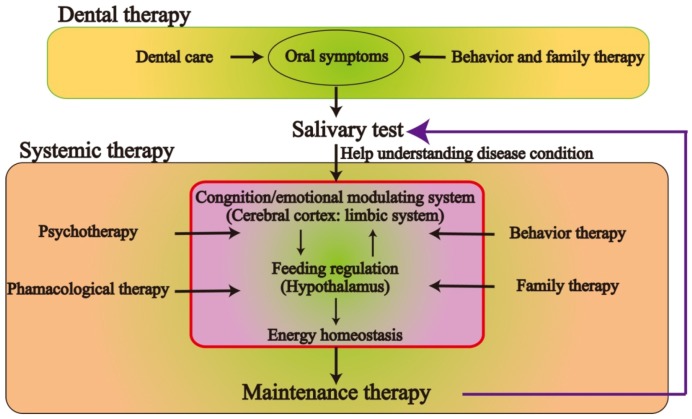
Schematic representation of the recommended treatment strategy for eating disorders. A multi-disciplinary team should manage each treatment stage for patients with eating disorders. Dental therapy teams are essential for initial detection of and pain control in oral manifestations. In addition, professional care provided by a dental practitioner will aid in the maintenance of oral function and hygiene control. However, systemic therapy requires behavioral and pharmacological approaches for control of energy homeostasis and psychopathology.

Salivary secretion is regulated by the central nervous system, which is related to food intake, emotional stress, and other factors*.* In addition, acinar cells in the salivary glands are a primary source of ghrelin that is produced and released in saliva, and the measurement of ghrelin in saliva may serve as a convenient alternative approach to the measurement of plasma ghrelin levels. Moreover, salivary cortisol levels might reflect the degree of stress experienced by eating disorder patients. Therefore, in adolescent and young patients with eating disorders, measurement of the salivary levels of the previously described substances and close collaboration with dentists may result in improved preventive care and positively influence the medical treatment strategy. Additional investigation of the brain–gut peptides and behavioral therapy, including a multidisciplinary approach with professional dental care, would result in greater treatment success for young patients with eating disorders. Figure 3 depicts the recommended treatment strategy for eating disorders. 

## 6. Conclusions

Eating disorders are difficult to treat, and patients chronically suffer from relapses. The etiology and pathophysiology of eating disorders are thought to involve a derangement of the feeding regulation network, including ghrelin, which causes an aberration in eating behavior. Ghrelin is also present in saliva, and measurement of ghrelin as well as of cortisol in the saliva may increase our understanding of the pathophysiological conditions present in eating disorder patients. 

Early detection and treatment can improve the likelihood of complete recovery, and patients with eating disorders are likely to exhibit several oral manifestations in the early stages of disease. The dentist and dental hygienist could be the first healthcare providers to observe these changes, and integrating them into an organized, multidisciplinary team would be highly beneficial for the treatment of eating disorder patients.
